# Spectral-domain OCT changes in retina and optic nerve in children with hypoxic–ischaemic encephalopathy

**DOI:** 10.1007/s00417-020-04996-y

**Published:** 2020-11-03

**Authors:** L. Grego, S. Pignatto, E. Busolini, N. Rassu, F. Samassa, R. Prosperi, C. Pittini, L. Cattarossi, Paolo Lanzetta

**Affiliations:** 1 Department of Medicine, Ophthalmology, University of Udine, Udine, Italy; 2 Clinica Oculistica, Azienda Sanitaria Universitaria Friuli Centrale (ASUFC), Ospedale Santa Maria della Misericordia, Piazzale Santa Maria della Misericordia, 15, 33100 Udine, Italy; 3 Patologia Neonatale - Azienda Sanitaria Universitaria Friuli Centrale (ASUFC) - Ospedale Santa Maria della Misericordia, Piazzale Santa Maria della Misericordia, 15, Udine, 33100 Italy

**Keywords:** Neonatal encephalopathy, Therapeutic hypothermia, Retina, Optic nerve

## Abstract

**Purpose:**

To evaluate the effect of neonatal hypoxic–ischaemic injury on the retina and the optic nerve and to correlate ocular damage with systemic parameters, laboratory tests, neurological imaging and therapeutic hypothermia at birth.

**Methods:**

Forty-one children with hypoxic–ischaemic encephalopathy (HIE) at birth (9.09 ± 3.78 years) and a control group of 38 healthy subjects (9.57 ± 3.47 years) were enrolled in a cohort study. The HIE population was divided into three subgroups, based on the degree of encephalopathy according to Sarnat score and the treatment with therapeutic hypothermia (TH): Sarnat score I not treated with hypothermia, Sarnat score II-III treated with TH and Sarnat score II-III not subjected to TH. Total macular thickness, individual retinal layers and peripapillary nerve fibre layer thickness were measured with spectral-domain optical coherence tomography. Clinical data of perinatal period of HIE children were collected: APGAR score, pH and base excess of funiculus blood at birth, apnoea duration, brain ultrasound, cerebral MRI ischaemic lesions and blood chemistry tests.

**Results:**

Children with Sarnat score I did not show a reduction of peripapillary nerve fibres and ganglion cell layer compared to the control group (*p* = 0.387, *p* = 0.316). Peripapillary nerve fibre layer was 109.06 ± 7.79 μm in children with Sarnat score II-III treated with TH, 108.31 ± 7.83 μm in subjects with Sarnat score II-III not subjected to TH and 114.27 ± 6.81 μm in the control group (*p* = 0.028, *p* = 0.007). Ganglion cell layer was thinner in children with Sarnat score II-III treated with TH (50.31 ± 5.13 μm) compared to the control group (54.04 ± 2.81 μm) (*p* = 0.01). Inner retinal layers damage correlated with C-reactive protein and lactate dehydrogenase increase, while higher levels of total bilirubin were protective against retinal impairment (*p* < 0.05). Cerebral oedema was related to peripapillary nerve fibre layer damage (*p* = 0.046).

**Conclusions:**

Thickness reduction of inner retinal layer and peripapillary nerve fibre impairment was related to encephalopathy severity. Ocular damage was associated with inflammation and cerebral oedema following hypoxic–ischaemic damage.

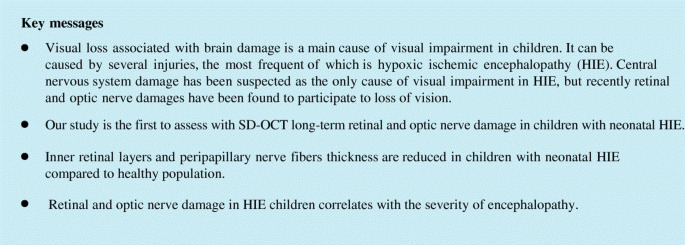

## Introduction

Neonatal hypoxic–ischaemic encephalopathy (HIE) is a clinical syndrome characterised by abnormal neurologic behaviour in the neonatal period due to inadequate blood flow and oxygen provision to the brain as a result of hypoxic–ischaemic events [1, 2]. Sarnat score divides HIE into three clinical stages (mild—grade I, moderate—grade II and severe—grade III-), based on different parameters: level of consciousness, neuromuscular control, complex reflexes, autonomic nervous system activity, seizures and electroencephalogram findings [3]. The standard of care for moderate and severe HIE includes therapeutic hypothermia (TH), which reduces core temperature to 33 °C–34 °C, with neuroprotective effects. Currently, two methods of TH are used: selective head cooling and whole-body cooling [4]. TH reduces the risk of mortality, neurodevelopment delay, blindness and cerebral palsy at 18 months. Although TH improves neurological outcomes in children with HIE, it does not provide complete protection: about 30% of treated children present different degrees of disability at 18 months of life [2, 5].

In developed countries, the most common cause of visual impairment is brain damage. It can be associated with several injuries, such as hypoglycaemia, epileptic seizures and hydrocephalus, although the most frequent cause is the hypoxic–ischaemic damage at birth [6]. Loss of visual function in HIE children have been primarily associated to hypoxic–ischaemic lesions of visual cortex in the occipital lobe, hence the term cortical blindness. This assertion has been questioned due to poor correlation between the degree of occipital cortex alterations, identified by computed tomography (CT) and nuclear magnetic resonance (MRI) and the severity of visual impairment [7–9]. Visual disability is strongly related to damaged visual associative areas, located in the temporal and parietal cortices, and visual attention pathways, located in the frontal lobe. White matter damage (periventricular leukomalacia and optical radiation impairments) contributes to visual dysfunction in HIE children [6] [10]. Currently, several studies are analysing retinal alterations associated with HIE in animal models, evaluating whether visual impairment could originate from a direct retinal damage as well as central nervous system lesions [11]. Inner retinal layer damage was detected in HIE mice, and TH seemed to have a protective effect on ganglion cell layer in asphyctic mice, preserving the retinal structure [11–13]. Few data are available in HIE children about retinal and optic nerve damage, always collected in aftermath of acute hypoxic insult. Retinal and choroidal thinning, associated with subretinal fluid, was detected with spectral-domain optical coherence tomography (SD-OCT) in HIE newborns [14]. Other findings identified with SD-OCT in asphytic children are perimacular cystoid spaces, ganglionar cell and peripapillary nerve fibre layer thinning [15]. So far, no studies evaluated retinal and optic nerve long-term outcomes after neonatal HIE.

The primary purpose of our study is to evaluate long-term retinal and peripapillary nerve fibre alterations with SD-OCT in children with HIE at birth, comparing them with a control group without neonatal asphyxia. The secondary purpose is to identify whether the ocular alterations correlate with systemic parameters at birth, laboratory data and radiological imaging of the central nervous system in perinatal period.

## Materials and methods

A cohort study was conducted at the Clinica Oculistica and at the Patologia Neonatale-Azienda Sanitaria Universitaria Friuli Centrale (ASUFC)-Ospedale Santa Maria della Misericordia, Udine, Italy, between February and September 2019. The study is adherent to the tenets of 1964 Helsinki declaration, and it is in accordance with the standards of the regional ethics committee (CEUR).

Forty-one children with diagnosis of HIE at birth (HIE group) and 38 healthy children (control group, CTL group), aged between 5 and 16 years, were enrolled. The population was born between January 2003 and September 2014.

Inclusion criteria for the HIE group were as follows: gestational age above 36 weeks, diagnosis of HIE performed at birth with neurologic examination and electroencephalogram, cycloplegic refractive error (calculated as spherical equivalent) between + 3 and − 3 dioptres. Exclusion criteria were congenital syndromes and severe disability resulting from the anoxic damage that precluded complete ophthalmological evaluation. Exclusion of children with a severe cerebral damage was due to the unavailability of a portable SD-OCT. Children not enrolled in the study presented higher degrees of mental and physical impairment, lowering significantly their compliance and precluding the use of traditional SD-OCT. No visual acuity cut off was set for HIE group.

Currently, recommendations prescribe TH in HIE children with Sarnat score II or III, while it is not recommended in newborn with Sarnat score I. In the Neonatal Intensive Care Unit of the Hospital of Udine, TH was available only from 2010. Therefore, children with Sarnat score II or III born before 2010 did not receive TH, while those with Sarnat score II or III born after 2010 were treated with TH. Thus, according to the degree of encephalopathy, availability and need of TH, HIE children were divided into three subgroups: Sarnat score II-III treated with hypothermia (SII-III/TH+), Sarnat score II-III not treated with hypothermia (SII-III/TH-) and Sarnat score I that did not require treatment with hypothermia (SI/TH-). Sarnat III children were few in our population (2 in the hypothermic subgroup, 3 in the non-hypothermic subgroup). Due to the low number, it was not possible to create a subgroup with only Sarnat III children. Therefore, we grouped children with Sarnat II and III for the study.

Inclusion criteria for the CTL group were gestational age above 36 weeks and cycloplegic refractive error (calculated as spherical equivalent) between + 3 and − 3 dioptres. Exclusion criteria were amblyopia, strabismus or other eye conditions and presence of systemic diseases.

One eye from each subject was randomly selected for analysis, using a random number generator. Enrolled children underwent complete ophthalmological evaluation and orthoptic examination. Best corrected visual acuity was measured with Snellen tables or E charts. Cyclopentolate eye drops were administered twice within an interval of 5 min, and cycloplegic refraction was measured using Retinomax K-plus3 autorefractor (Righton), approximately 30 min after the second eye drop. Spherical equivalent (calculated as spherical error + half cylinder error) was obtained from the refractive error value for each eye. Slit-lamp biomicroscopy of the anterior segment and indirect ophthalmoscopy of the fundus were conducted.

SD-OCT (Heidelberg Engineering, Dossenheim, Germany) was performed after pupil dilation. Three maps of the macular region centred on the fovea, with 25 scans spaced 237 μm and a total area of 5700 × 5700 μm, were obtained. The highest quality map was chosen for analysis. Quality score was automatically assigned by SD-OCT software, expressed as signal-to-noise ratio in decibels. Scans over 20 dB were considered of high quality. Each scan of the 25 macular scans was automatically segmented to single out the profile and thickness of each individual retinal layer. Automatic segmentation of each scan was checked and manually corrected by two independent observers (L.G., S.P.). The following retinal layers were measured: macular nerve fibre layer (MNFL), ganglion cell layer (GCL), inner plexiform layer (IPL), inner nuclear layer (INL), outer plexiform layer (OPL), outer nuclear layer (ONL), retinal pigment epithelium (RPE), inner retinal layers (IRL) and outer retinal layers (ORL). Early Treatment Diabetic Retinopathy Study (ETDRS) grid evaluated the macular and individual retinal layers thickness, each divided into three zones: average thickness in the central square millimetre; average thickness of superior, temporal, inferior and nasal segments of the ring with 3 mm diameter and average thickness of superior, temporal, inferior and nasal segments of the ring of 6 mm diameter. For the purposes of our study, we considered the average of values of the SD-OCT ETDRS grid with 3 mm diameter (3 mm), 6 mm diameter (6 mm) and the mean of all segments of ETDRS grid (total).

Three circular scans of the peripapillary nerve fibre layer (PNFL) with a diameter of 3.5 mm were obtained. PNFL scans with the best image quality were chosen for the analysis. SD-OCT software automatically calculates PNFL thickness in the central, temporal, superior-temporal, superior-nasal, nasal, inferior-nasal and inferior-temporal sectors. Average values were used for the study.

Data regarding the perinatal period were obtained from the clinical data management system and the medical records for the HIE group: Sarnat score; gestational age; weight at birth; APGAR score at 1, 5 and 10 min of life; pH and base excess in funiculus blood at birth; arterial blood oxygen pressure, carbon dioxide pressure and lactates measured at the first sampling performed by entering in the Neonatal Intensive Care Unit; brain ultrasound alterations (hyperechogenicity of periventricular white matter, impaired white/grey matter differentiation and/or slit-like ventricles); cerebral MRI ischaemic lesions; apnoea duration and overall duration of respiratory assistance and latency of visual evoked potentials flashes (VEP) performed within the first 10 days of life. In addition, blood chemistry tests of the first 3 days were collected, including red blood cells, white blood cells, platelets, haemoglobin, haematocrit, mean corpuscular volume (MCV), mean corpuscular haemoglobin concentration (MCHC), C-reactive protein (CRP), urea nitrogen, creatinine, lactate dehydrogenase (LDH), serum glutamic oxaloacetic transaminase (SGOT), serum glutamic pyruvate transaminase (SGPT), total and direct bilirubin and albumin. For statistical analysis, the mean value of blood chemistry tests of the first three days was considered. Blood chemistry values for each day were not available for all patients, as they were performed according to the clinical status of HIE children. For CRP, the values of day 4 and 5 were evaluated in order to analyse the evolution over time.

Statistical analysis was conducted using SPSS Statistic version 22 (IBM Corporation). Normality of continuous variables was evaluated with the Shapiro–Wilk test. One-way ANOVA followed by multiple comparisons of Bonferroni and Kruskal Wallis tests followed by post hoc test were used to identify the differences in total retinal thickness, individual retinal layers thickness and PNFL between the encephalopathic patients and CTL group, respectively for normal and non-normal variables. Pearson and Spearman tests were used for the correlation of continuous systemic parameters (blood chemistry tests mean value of first three days of life) with MNFL, GCL and PNFL. It was not possible to perform repeated measures ANOVA or Friedman test to look for a correlation between haematochemical tests and retinal thickness over time, due to blood test data not being available for each day in all patients. *T* test and Mann–Whitney test were used for the correlation of dichotomous systemic parameters and MNFL, GCL and PNFL. *p* value < 0.05 was considered statistically significant.

## Results

### Characteristics of the population

Forty-one children with HIE at birth (mean age of 9.09 ± 3.78 years) were enrolled (HIE group). The population was divided into three subgroups, based on degree of encephalopathy and treatment with TH:

- SII-III/TH+ subgroup: 18 children with Sarnat score II-III treated with TH (mean age of 6.14 ± 0.91 years, 10 males and 8 females)
- SII-III/TH- subgroup: 12 children with Sarnat score II-III, no TH (mean age of 13.05 ± 2.62 years, 7 males and 5 females)
- SI/TH- subgroup: 11 children with Sarnat score I, no TH (mean age of 9.6 ± 3.67 years, 6 males and 5 females)

**Fig. 1:**
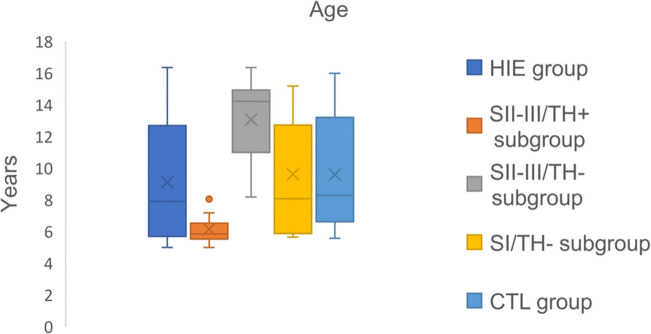
Age distribution of enrolled population

Thirty-eight healthy children (13 males and 25 females) with a mean age of 9.57 ± 3.47 years were enrolled as the CTL group. Age was not significantly different between the HIE group and the CTL group (*p* = 0.426), whereas this was significantly different in the subgroups (*p* < 0.05) (Fig. 1). Age difference between subgroups can be explained due to TH being performed based on the severity of HIE and TH being available in the Neonatal Intensive Care Unit from 2010: children of SII-III/TH- subgroup were born between 2003 and 2009 when TH was not available, children of SII-III/TH+ subgroup were born between 2010 and 2014 and where treated with TH, while the children of SI/TH -, that did not need TH, were born between 2003 and 2014.

Gestational age, birth weight, APGAR score, pH and base excess in funiculus blood at birth, arterial blood lactates and duration of apnoea are reported in Table 1. Mean PCR values of the HIE group in the first 5 days of life were the following: 3.52 ± 8.23 mg/l in the first day, 9.66 ± 10.68 mg/l in the second day, 20.97 ± 27.60 mg/l in the third day, 33.2 ± 57.56 mg/l in the fourth day and 27.96 ± 31.59 mg/l fifth day. LDH mean value of the HIE group in the first 3 days was as follows: 2438.64 ± 1187.56 U/l first day, 3043.5 ± 2333.53 U/l second day and 1265.6 ± 234.64 U/l third day of life.

**Table 1:** Baseline characteristic of the population

	Number of patients	Age (years)	Weight at birth (grams)	Gestational age (weeks)	APGAR score 1 min	APGAR score 5 min	APGAR score 10 min	pH funiculus blood at birth	Base excess in funiculus blood	Arterial blood lactates (mEq/l)	Duration apnoea (minutes)
HIE group	41	9.09 ± 3.78	3314.24 ± 610.19	39.17 ± 2.24	2.78 ± 2.08	5.17 ± 1.77	6.77 ± 1.66	7.02 ± 0.17	−14.14 ± 6.03	18.04 ± 35.98	5.68 ± 5.78
SII-III/TH+ subgroup	18	6.14 ± 0.91	3239 ± 337.98	39.13 ± 1.66	2.77 ± 2.07	4.94 ± 1.66	6.31 ± 1.77	7.01 ± 0.19	−13.78 ± 5.93	7.15 ± 5.03	6.41 ± 7.54
SII-III/TH- subgroup	12	13.05 ± 2.62	3376 ± 787.44	39.67 ± 2.81	2.83 ± 2.72	5.17 ± 2.37	6.7 ± 1.7	6.99 ± 0.14	−18.05 ± 6.23	47.7 ± 61.27	4.58 ± 3.99
SI/TH- subgroup	11	9.6 ± 3.67	3418 ± 775.65	38.71 ± 2.78	2.73 ± 1.35	5.55 ± 1.21	7.67 ± 1.12	7.08 ± 0.18	− 10.99 ± 4.25	6.9 ± 6.12	5.73 ± 4.43
CTL group	38	9.57 ± 3.47	3240.96 ± 346.82	39.61 ± 1.14	NA	NA	NA	NA	NA	NA	NA

All children had a best corrected visual acuity of 10/10 at Snellen chart or E chart. One subject of the SI/TH- subgroup had a microesotropy and absence of stereopsis to Lang I and II tests, while the other patients did not present alterations to orthoptic evaluation. Spherical equivalent was similar in the groups: the HIE group 1.05 ± 0.67, SII-III/TH+ subgroup 1.26 ± 0.73, SII-III/TH- subgroup 1.07 ± 0.78, SI/TH- subgroup 1.08 ± 0.57 and CTL group 0.78 ± 1.81 (*p* > 0.05). No anterior segment alterations were found. Optic nerve drusen were detected in a single patient of the SII-III/TH- subgroup; therefore, his thickness of the PNFL was not considered in the analyses. No other ocular fundus or optic disc alterations were found.

### Comparison of retinal thickness between HIE children and CTL group

**Fig. 2:**
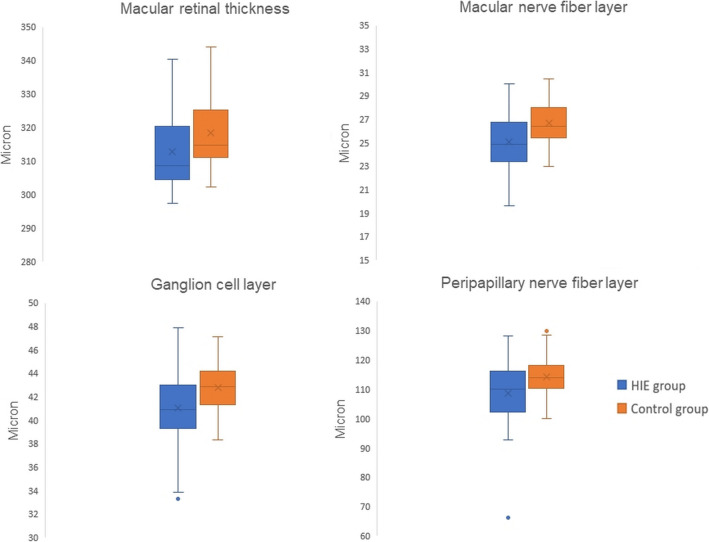
Comparison of retinal thicknesses (total macular thickness, macular nerve fibre layer, ganglion cells layer and peripapillary nerve fibre layer) between the HIE group and control group

Macular retinal thickness 3 mm, 6 mm and total were lower in the HIE group compared to the CTL group (*p* = 0.001, *p* = 0.039, and *p* = 0.020). Analysis of individual retinal layers revealed a difference in MNFL3 mm, 6 mm and total (*p* = 0.001, *p* = 0.005, and *p* = 0.001) and GCL 3 mm, 6 mm and total (*p* = 0.002, *p* = 0.035, and *p* = 0.008), which were reduced in HIE children. IPL 3 mm and total were lower in the HIE group than in the CTL group (*p* = 0.02, *p* = 0.035). IPL 6 mm was not significantly reduced in subjects within the HIE group (*p* = 0.077). Measurement of the inner retinal layers 3 mm, 6 mm and total confirmed that the damage in HIE children affects mainly the inner retina (*p* = 0.007, *p* = 0.05, and *p* = 0.024), while outer retinal layers thickness 3 mm, 6 mm and total was not different between the HIE and CTL groups (*p* = 0.568, *p* = 0.393, and *p* = 0.463). Finally, PNFL thickness was lower in the HIE group than in the CTL group (*p* = 0.008) (Tables 2 and 3 and Fig. 2).

**Table 2:** Total retinal thickness and individual retinal layer thickness for groups enrolled in the study

		HIE group (mean ± standard deviation)	SII-III/TH+ subgroup (mean ± standard deviation)	SII-III/TH- subgroup (mean ± standard deviation)	SI/TH- subgroup (mean ± standard deviation)	CTL group (mean ± standard deviation)
Total retinal thickness (μm)	3 mm	312.74 ± 11.29^*^	311.30 ± 11.04^*^	313.27 ± 8.54^*^	314.53 ± 14.68^*^	328.42 ± 18.14
	6 mm	301.26 ± 11.03^*^	302.90 ± 8.75	299.66 ± 9.57	300.34 ± 15.73	307.09 ± 10.89
	Total	312.74 ± 11.29^*^	311.30 ± 11.04^*^	313.26 ± 8.54	314.53 ± 14.68	318.49 ± 10.18
Macular nerve fibre layer (μm)	3 mm	19.73 ± 1.56^*^	19.24 ± 1.46^*^	20.25 ± 1.12	19.98 ± 1.98	21.04 ± 1.37
	6 mm	33.64 ± 3.98^*^	33.47 ± 3.58^*^	33.50 ± 2.92^*^	34.06 ± 5.65	36.11 ± 3.17
	Total	25.08 ± 2.40^*^	24.70 ± 2.28^*^	25.30 ± 1.62	25.46 ± 3.29	26.69 ± 1.79
Ganglion cell layer (μm)	3 mm	51.20 ± 4.85^*^	50.31 ± 5.13^*^	52.81 ± 2.49	50.91 ± 6.14	54.04 ± 2.81
	6 mm	37.28 ± 2.87^*^	37.89 ± 2.55	36.92 ± 2.41	36.68 ± 3.78	38.68 ± 2.48
	Total	41.08 ± 3.18^*^	40.83 ± 3.41	41.62 ± 1.97	40.90 ± 4.01	42.76 ± 2.17
Inner plexiform layer (μm)	3 mm	41.92 ± 3.34^*^	41.50 ± 3.77	42.27 ± 1.58	42.23 ± 4.14	43.43 ± 2.09
	6 mm	30.30 ± 2.26	30.86 ± 1.97	29.94 ± 2.01	29.80 ± 2.88	31.23 ± 1.89
	Total	34.43 ± 2.23^*^	34.41 ± 2.31	34.36 ± 1.64	34.54 ± 2.81	35.38 ± 1.62
Inner nuclear layer (μm)	3 mm	41.12 ± 2.89	41.71 ± 3.61	40.06 ± 1.89	41.30 ± 2.29	41.98 ± 2.72
	6 mm	35.53 ± 2.03	36.33 ± 2.04	34.60 ± 1.59	35.23 ± 2.08	35.90 ± 2.55
	Total	36.07 ± 2.20	36.64 ± 2.64	35.06 ± 1.51	36.23 ± 1.78	36.46 ± 2.12
Outer plexiform layer (μm)	3 mm	34.16 ± 4.05	36.31 ± 4.38	32.83 ± 3.43	32.11 ± 2.25	33.88 ± 4.74
	6 mm	27.64 ± 1.96	28.33 ± 2.15	27.21 ± 2.01	26.98 ± 1.22	27.61 ± 2.51
	Total	30.54 ± 2.86	32.02 ± 3.06	29.41 ± 2.50	29.35 ± 1.69	30.19 ± 3.37
Outer nuclear layer (μm)	3 mm	68.35 ± 7.80	65.90 ± 7.85	69.54 ± 7.46	71.05 ± 7.56	69.90 ± 7.13
	6 mm	59.24 ± 5.68	59.01 ± 5.55	59.17 ± 5.92	59.70 ± 6.16	59.53 ± 5.17
	Total	66.24 ± 6.58	64.71 ± 6.66	67.16 ± 6.28	67.75 ± 6.85	67.41 ± 6.05
Retinal pigment epithelium (μm)	3 mm	13.18 ± 1.20	13.14 ± 1.16	13.21 ± 1.34	13.23 ± 1.22	13.23 ± 1.20
	6 mm	12.16 ± 0.99	12.06 ± 0.91	12.33 ± 1.18	12.14 ± 0.97	12.22 ± 1.01
	Total	13.09 ± 1.03	13.02 ± 1.04	13.17 ± 1.18	13.13 ± 0.92	13.12 ± 1.02
Inner retinal layers (μm)	3 mm	256.55 ± 13.34^*^	255.00 ± 14.35	257.77 ± 8.81	257.77 ± 16.41	264.14 ± 10.77
	6 mm	223.61 ± 11.16^*^	225.89 ± 9.01	221.21 ± 9.77	222.50 ± 15.42	229.13 ± 10.41
	Total	233.28 ± 11.13^*^	233.15 ± 11.17	232.59 ± 8.24	234.24 ± 14.39	238.65 ± 9.40
Outer retinal layers (μm)	3 mm	80.01 ± 2.03	79.36 ± 1.47	80.63 ± 2.14	80.39 ± 2.52	80.26 ± 1.84
	6 mm	77.45 ± 1.43	76.88 ± 0.89	78.08 ± 1.59	77.70 ± 1.69	77.86 ± 1.93
	Total	79.69 ± 1.72	79.00 ± 1.21	80.37 ± 1.88	80.07 ± 1.98	79.98 ± 1.78
Peripapillary circular scans of nerve fibres (μm)		108.68 ± 10.70^*^	109.06 ± 7.79^*^	108.31 ± 7.83^*^	108.40 ± 16.76	114.27 ± 6.81

^*^Statistically significant over the control group

**Table 3:** Standard error of the mean (SEM) and confidence interval (CI) for statistically significant value

		HIE group		SII-III/TH+ subgroup		SII-III/TH- subgroup		SI/TH- subgroup		CTL group	
		mean ± SEM	IC 95%	mean ± SEM	IC 95%	mean ± SEM	IC 95%	mean ± SEM	IC 95%	mean ± SEM	IC 95%
Total retinal thickness (μm)	3 mm	312.74 ± 1.76^*^	309.18–316.31	311.30 ± 2.60^*^	305.81–316.79	313.27 ± 2.47^*^	307.84–318.70	314.53 ± 4.43^*^	304.67–324.38	328.42 ± 2.94	322.46–334.39
	6 mm	301.26 ± 1.72^*^	297.78-304.75	302.90 ± 2.06	298.55–307.25	299.66 ± 2.76	293.58–305.75	300.34 ± 4.74	289.77–310.91	307.09 ± 1.77	303.52–310.68
	Total	312.74 ± 1.76^*^	309.17-316.31	311.30 ± 2.60^*^	305.81- 316.79	313.26 ± 2.47	307.84–318.69	314.53 ± 4.43	304.67–324.38	318.49 ± 1.65	315.14–321.84
Macular nerve fibre layer (μm)	3 mm	19.73 ± 0.24^*^	19.24–20.22	19.24 ± 0.34^*^	18.51–19.96	20.25 ± 0.32	19.53–20.96	19.98 ± 0.60	18.65–21.31	21.04 ± 0.22	20.60–21.50
	6 mm	33.64 ± 0.62^*^	32.38–34.90	33.47 ± 0.84^*^	31.69–35.25	33.50 ± 0.84^*^	31.64–35.35	34.06 ± 1.70	30.27–37.86	36.11 ± 0.51	35.06–37.15
	Total	25.08 ± 0.37^*^	24.32–25.84	24.70 ± 0.54^*^	23.56–25.83	25.30 ± 0.47	24.26–26.33	25.46 ± 0.99	23.25–27.68	26.69 ± 0.29	26.10–27.28
Ganglion cell layer (μm)	3 mm	51.20 ± 0.76^*^	49.67–52.73	50.31 ± 1.21^*^	47.76–52.86	52.81 ± 0.72	51.23–54.40	50.91 ± 1.85	46.78–55.03	54.04 ± 0.46	53.11–54.96
	6 mm	37.28 ± 0.45^*^	36.37–38.19	37.89 ± 0.60	36.62–39.15	36.92 ± 0.70	35.38–38.45	36.68 ± 1.14	34.15–39.22	38.68 ± 0.40	37.86–39.49
	Total	41.08 ± 0.50^*^	40.08–42.09	40.83 ± 0.80	39.14–42.53	41.62 ± 0.57	40.37–42.87	40.90 ± 1.21	38.20–43.59	42.76 ± 0.35	42.05–43.47
Inner plexiform layer (μm)	3 mm	41.92 ± 0.52^*^	40.87–42.97	41.50 ± 0.89	39.62–43.37	42.27 ± 0.46	41.27–43.27	42.23 ± 1.25	39.45–45.01	43.43 ± 0.34	42.74–44.12
	6 mm	30.30 ± 0.35	29.59–31.02	30.86 ± 0.46	29.88–31.84	29.94 ± 0.58	28.66–31.21	29.80 ± 0.87	27.86–31.73	31.23 ± 0.31	30.61–31.85
	Total	34.43 ± 0.35^*^	33.72-35.14	34.41 ± 0.54	33.26–35.56	34.36 ± 0.47	33.32–35.40	34.54 ± 0.85	32.65–36.42	35.38 ± 0.26	34.85–35.91
Inner retinal layers (μm)	3 mm	256.55 ± 2.08^*^	252.34–260.77	255.00 ± 3.38	247.87–262.13	257.77 ± 2.54	252.17–263.37	257.77 ± 4.95	246.75–268.80	264.14 ± 1.75	260.60–267.69
	6 mm	223.61 ± 1.74^*^	220.09–227.13	225.89 ± 2.12	221.40–230.37	221.21 ± 2.82	215.00–227.41	222.50 ± 4.65	212.14–232.86	229.13 ± 1.69	225.71–232.55
	Total	233.28 ± 1.74^*^	229.77-236.79	233.15 ± 2.63	227.60–238.70	232.59 ± 2.38	227.36–237.83	234.24 ± 4.34	224.58–243.91	238.65 ± 1.52	235.56–241.74
Peripapillary circular scans of nerve fibres (μm)		108.68 ± 1.69^*^	105.25–112.09	109.06 ± 1.84^*^	105.19–112.94	108.31 ± 2.36^*^	103.05–113.58	108.40 ± 5.05	97.14–119.66	114.27 ± 1.10	112.03–116.51

^*^Statistically significant over the control group

### Comparison of retinal thickness between HIE subgroups and CTL group

**Fig. 3:**
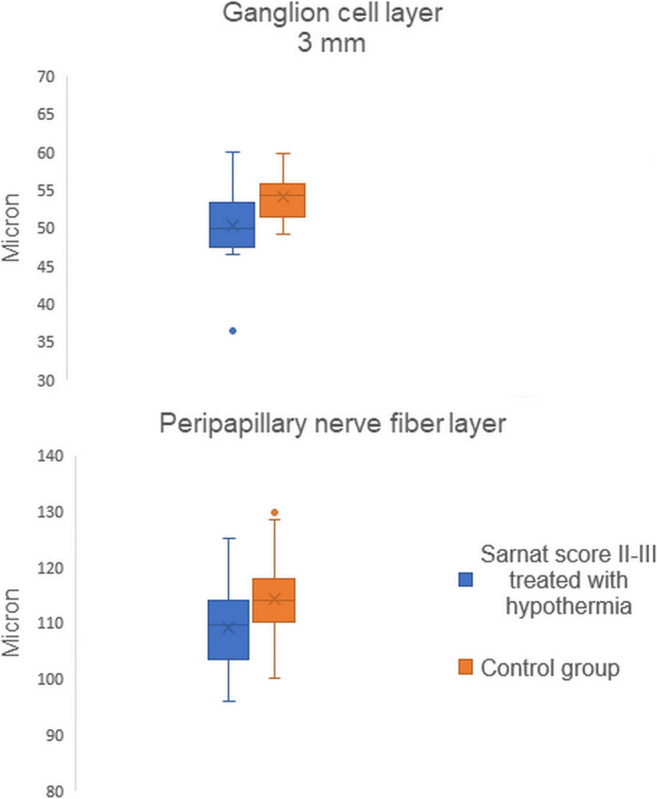
Comparison of ganglion cells layer and peripapillary nerve fibre layer thickness between the Sarnat II-III children subjected to hypothermia and control group

Macular retinal thickness 3 mm was lower in the SII-III/TH+, SII-III/TH- and SI/TH- subgroups compared to the CTL group (*p* = 0.001, *p* = 0.021, and *p* = 0.045). Furthermore, total macular retinal thickness was reduced in the SII-III/TH+ subgroup compared to the CTL group (*p* = 0.008). Analysis of individual retinal layers revealed a lower value of MNFL 3 mm, 6 mm and total in the SII-III/TH+ subgroup compared to the CTL group (*p* = 0.001, *p* = 0.014, and *p* = 0.010); moreover, MNFL 6 mm were reduced in the SII-III/TH- subgroup compared to the CTL group (*p* = 0.034). Hypothermic HIE children showed a reduction of GCL 3 mm compared to the CTL groups (*p* = 0.01). Finally, PNFL thickness was lower in the SII-III/TH+ and SII-III/TH- subgroups than in the CTL group (*p* = 0.028, *p* = 0.007). Differences in retinal thickness and peripapillary nerve fibre layers in the SII-III/TH+, SII-III/TH- and SI/TH- subgroups were not significant. The values of retinal thickness are reported in Table 2 and Fig. 3. Standard error of the mean and confidence intervals are shown in Table 3.

### Correlations between retinal thickness measured with SD-OCT and systemic parameters

**Fig. 4:**
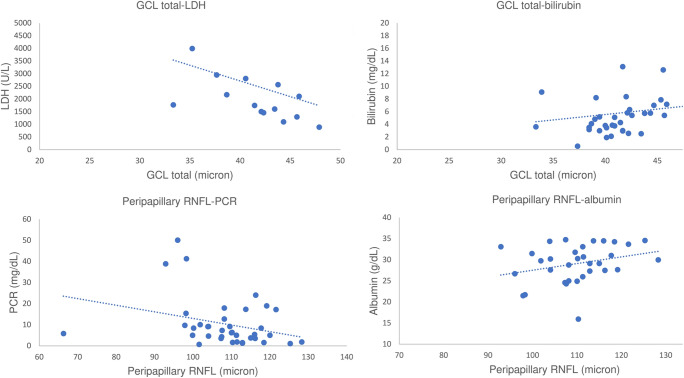
Correlation between GCL and PNFL with systemic parameters in the HIE group

**Fig. 5:**
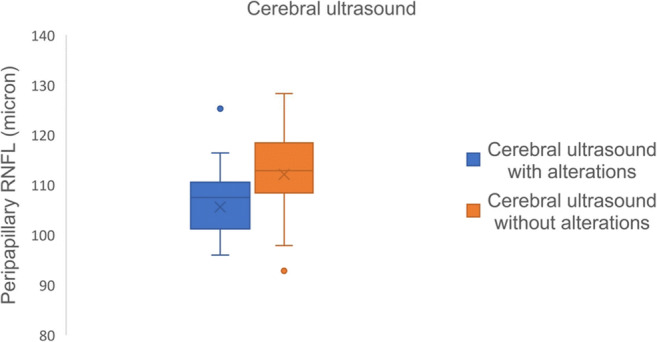
Distribution of peripapillary retinal thickness in HIE children with or without cerebral ultrasound alterations

In the HIE group, GCL 3 mm correlated positively with total bilirubin (*p* < 0.05) and negatively with LDH and PCR (*p* < 0.01). Greater thickness of GCL total was associated with higher total bilirubin and lower LDH (*p* < 0.05). GCL 6 mm does not correlate with systemic parameters. Higher thickness of PNFL correlated with higher albumin (*p* < 0.01) and lower PCR (*p* < 0.05) (Fig. 4). Furthermore, lower PNFL thickness was observed in children with alterations at cerebral ultrasound (hyperechogenicity of periventricular white matter, impaired white/grey matter differentiation and/or slit-like ventricles) (*p* = 0.046) (Fig. 5).

APGAR score, pH and base excess in funiculus blood at birth, apnoea duration, cerebral MRI ischaemic lesions and PEV latency did not have a statistically significant correlation with retinal layers thickness, neither for total population, nor for subgroups (*p* > 0.05).

## Discussion

To the best of our knowledge, this is the first study that evaluates long-term retinal damage with SD-OCT in children with hypoxic–ischaemic encephalopathy at birth, stratifying the population according to the severity of the hypoxic insult and the hypothermic treatment for HIE.

We studied HIE children and we found that the thicknesses of PNFL, MNFL, GCL and IPL were reduced, without any subsequent visual functional damage. Among the subgroups, thickness reduction of inner retinal layers can be found in patients with Sarnat score II-III compared to healthy subjects, regardless of any therapeutic hypothermic treatment. Experimental studies on murine models report damage to the inner retinal layers with HIE (ganglion cell layer, inner plexiform layer and inner nuclear layer) [11–13]. Mangalesh et al. found ganglion cell and peripapillary nerve fibre layer impairment assessing HIE children with SD-OCT within the first two weeks of hypoxic–ischaemic insult [15]. Osborn et al. showed greater susceptibility of inner retina to lack of oxygen compared to outer retinal layers. Outer retinal layers and photoreceptors receive blood supply directly from the central retinal artery and indirectly from the choriocapillaris, and this double blood supply is protective against hypoxic–ischaemic damage. Inner retinal layers, on the other hand, are vascularized the by central retina artery alone and, in 30% of the population, by the cilioretinal artery which supplies the macula. Central retinal artery occlusion damages mainly the inner retinal layers, while outer retinal layers damage is related to choriocapillaris ischemia or to the separation of the photoreceptor layer from the retinal pigment epithelium [16].

For some statistically significant values of retinal thickness, there was a minimal overlap of confidence intervals and of standard error of mean [17]. This could affect clinical significance of our results, although the overlaps were less than 1 μm. In addition, clinical significance of our study was difficult to assess, due to absence of similar analysis and for the limited population enrolled.

In many studies, visual loss in HIE children was attributed to cortical visual impairment, due to trans-synaptic retrograde damage following occipital cortex impairment. However, this assertion has been questioned, because visual damage frequently did not correlate with the severity of magnetic resonance signs of injury [13]. Chan et al., evaluating rats with neonatal hypoxia ischemia through magnetic resonance, demonstrated that damage to retinocollicular and retinogeniculate pathways occurs due to an anterograde process starting from the eye. Although both eyes and visual pathways were damaged in HIE rats, the contribution of said damage to visual impairment was not defined [18]. Mangalesh et al. found an association between retinal defects and MRI alterations evaluating 8 HIE children: newborns with low-grade MRI changes had no retinal findings, children with intermediate MRI damage had subretinal fluid or macular cystic spaces, while children with more severe MRI alterations had parafoveal cystoid spaces and loss of GCL/RNFL in the macula. Moreover, the author pointed out that children with intermediate MRI damage showed a different kind of retinal damage, suggesting a possible primitive eye injury independent from brain damage [15]. In our study, the enrolled population did not present visual impairment, as we measured a best corrected visual acuity of 10/10 in all patients. Then, we evaluated if there was a correlation between the reduction of retinal thickness in SD-OCT and MRI ischaemic areas. This correlation was not detected, suggesting a possible primary retinal damage in HIE children. In any case, we cannot exclude a retrograde or anterograde damage component, because OCT retinal changes were in the order of magnitude of microns, while conventional magnetic resonance has a resolution of 1 mm [19].

In the subgroups analysis, a statistically significant difference of inner retinal layers thickness and peripapillary nerve fibre was observed between HIE children with Sarnat score II-III with or without hypothermic treatment and healthy patients. There was no difference between HIE patients with Sarnat score I and healthy children. Retinal thickness was not different in children with Sarnat score II-III who did or did not receive hypothermia suggesting that TH was not protective against retinal damage. In murine models, retinal damage correlated with hypoxia severity at birth, with a progressive reduction of inner retinal layers thickness as hypoxia severity increased [11]. Rey-Funes et al. showed that therapeutic hypothermia was protective against inner retinal damage, while in rats with perinatal asphyxia that were not subjected to therapeutic hypothermia, there was an alteration of the ganglion cell layer, no neurodegenerative aspects of this layer were observed in the hypothermic group [12]. The discrepancy with the outcomes of the present study may be due to differences with animal models and to selection bias. Only patients able to undergo a complete ophthalmological evaluation were enrolled, thus excluding those with severe functional disabilities following hypoxic–ischaemic encephalopathy. Patients with Sarnat score II or III not subjected to hypothermia that were excluded from the study may possibly have greater damage to the inner retina. This may explain the absence of differences of inner retinal layers thickness between the SII-III/TH- subgroup and children treated with hypothermia. This was not an issue in rat studies, where the retinal thicknesses of all animals were evaluated. Furthermore, hypoxic conditions at birth may vary for each child: apnoea duration, comorbidities and causes of HIE may affect the outcome of ocular and cerebral damage. In experimental models, hypoxic–ischaemic damage was standardised for all the enrolled animals, producing homogeneous results in the samples.

Several correlations emerged by comparing retinal thickness and peripapillary nerve fibre layer with systemic parameters related to the severity of neonatal hypoxic–ischaemic damage. This emphasises how ocular damage fits into a complex damage system resulting from HIE.

C-reactive protein is an acute phase protein whose levels increase with the severity of encephalopathy. Therapeutic hypothermia results in a slower increase in CRP, but cooled children presented a later and higher peak of this value. A greater increase characterised patients with multiorgan failure [20]. LDH values are higher in patients with grey matter abnormalities, rendering it a predictive biomarker of neurodevelopmental prognosis in HIE children [21]. In our population, mean values of LDH and CRP levels in the first three days of life were higher in children with greater damage to ganglion cells layer, while peripapillary nerve fibre reduction was related only to CRP increase. CRP mean values increased progressively in HIE children during the first 4 days of life, while it began to fall on the fifth day. Unfortunately, the mean values of single days were distorted by the lack of many values: the measurement was no longer repeated in children with an early normalisation or no increase of CRP, resulting in invalid higher values. A similar consideration can be made for LDH, which starts to fall on the third day of life. The reason of CRP increase in HIE is not yet fully known. One of the crucial but not studied mechanisms of secondary neuronal damage after hypoxic–ischaemic damage is inflammation [22, 23]. Two phases of CNS damage can be identified in neonatal HIE. The first is related to acute hypoxia which involves cellular energy failure, acidosis, release of glutamate, accumulation of intracellular calcium, lipid peroxidation and nitric oxide neurotoxicity causing cell death. This phase is affected by apnoea duration and it causes pH and base excess alterations. The second damage phase occurs 6 to 48 h after the hypoxic insult, it is related to the reperfusion and it is a reaction to the primary insult. In the secondary phase, the free radical production and nitric oxide formation leads to mitochondria dysfunction, and circulatory and endogenous inflammatory cells/mediators also contribute to brain injury [24, 25]. Association between retinal thinning (ganglion cell and peripapillary nerve fibre layer) and increased inflammation (CRP and LDH) could be related to reperfusion damage. However, our data are too weak to define a certain association with the second damage phase 6–48 h after the primary anoxic insult. During embryogenesis, the eye develops from a diencephalic extroflexion; therefore, retinal tissues react to hypoxic damage in a way similar to brain tissues but are more resistant to ischaemic insult than the central nervous system. Only few minutes of cerebral ischaemia may cause irreversible damage, while retinal tissues in primates may endure for over 100 min after a central retinal artery occlusion before irreversible damage occurs. Retinal resistance to ischaemia may be related to neuroglobins, proteins similar to haemoglobin and myoglobin that can be found especially in photoreceptors. These cells can obtain energy even through anaerobic metabolism [16]. Photoreceptors also possess numerous mitochondria, which can increase in size with hypoxia, making them more resistant to apoptosis [11]. These protective mechanisms could explain the absence of correlation of retinal damage with the duration of apnoea, pH and excess base and index related to the first phase of damage.

In HIE children, higher total bilirubin is associated with greater ganglion cell thickness. Bilirubin is formed by the degradation of heme. Low bilirubin concentration is protective towards the central nervous system, acting as an antioxidant, while it is neurotoxic at high levels [26, 27]. Total bilirubin values are lower in HIE children compared to healthy newborns, probably because it is consumed for its antioxidant effect in newborns with severe oxidative stress [28]. Its protective effect has been also evaluated in the eye: bilirubin antioxidant effect has been assessed in retinopathy of prematurity, but results disagree in the studies [29–31]. In our study, a lower ganglion cell damage was present in children with higher levels of bilirubin, which could exert its protective antioxidant effect on free radicals produced in the retina during the reperfusion phase.

Albumin maintains osmotic blood pressure and transports different molecules in the bloodstream. It also possesses an antioxidant effects, playing a role in neuron survival during development, especially in children with suffering at birth [32]. Albumin administration in HIE children reduces cerebral oedema and improves symptoms associated to encephalopathy [33]. Cerebral oedema can be assessed by cerebral ultrasound, a non-invasive and low-cost method that allows central nervous system evaluation. HIE children compared to the CTL group present an increase in white matter echogenicity compared to to grey matter due to cerebral oedema and possible necrosis of nervous tissue [34]. Findings such as slit-like ventricles are also suggestive of cerebral oedema [35]. In our study, lower albumin and cerebral ultrasound signs of cerebral oedema correlate with lower peripapillary nerve fibre layer thickness. We can hypothesise that the damage is related to the presence of cerebral oedema at birth, resulting in deterioration and reduction of optic nerve fibres.

Our study has numerous limitations: non-homogeneity of ages in the comparison between subgroups, exclusion of patients with severe outcomes deriving from HIE, non-execution of tests for assessment of visual quality (microperimetry, visual field) and electrophysiology tests because the smaller patients would not have the necessary collaboration, the assessment of ischaemic brain lesions regardless their location.

## Conclusion

In our study, children with neonatal moderate and severe HIE had a slight reduction in the inner retinal layer thickness as shown in murine models, which did not correspond to a visual acuity impairment. These results were found in a population with neonatal HIE without systemic or neurologic long-term sequelae. Hypothermic treatment did not appear to be protective against ocular hypoxic–ischaemic insults, although this may be due to a selection bias. The damage to the inner retinal layers is related to higher CRP and LDH levels in the early days of life. Cerebral oedema may lead to peripapillary nerve fibre layer impairment. HIE subjects have a lower functional reserve of ganglion cells and nerve fibre layer, which could make them more susceptible in adulthood to diseases that damage inner retinal layers, such as glaucoma or optic neuritis. Further studies are needed to understand the pathophysiology of retinal damage in subjects with neonatal hypoxic–ischaemic injury. It would be interesting to assess possible retinal sensitivity damage (e.g. with microperimetry or electroretinography) related to ganglion cell and peripapillary nerve fibre layer undetectable with visual acuity testing alone. The identification of subclinical retinal damage in HIE children without long-term sequelae makes necessary the assessment of children with hypoxic–ischaemic damage outcomes to evaluate their eye impairment and whether it correlates with visual acuity impairment.

## Abbreviations

HIE: hypoxic–ischaemic encephalopathy
TH: therapeutic hypothermia
MRI: nuclear magnetic resonance
CT: computed tomography
SD-OCT: spectral-domain optical coherence tomography
HIE group: hypoxic–ischaemic encephalopathy group
CTL group: control group
SII-III/TH+ subgroup: Sarnat score II-III and treated with hypothermia
SII-III/TH- subgroup: Sarnat score II-III and not treated with hypothermia
SI/TH- subgroup: Sarnat score I and not treated with hypothermia
MNFL: macular nerve fibre layer
GCL: ganglion cell layer
IPL: inner plexiform layer
INL: inner nuclear layer
OPL: outer plexiform layer
ONL: outer nuclear layer
RPE: retinal pigment epithelium
IRL: inner retinal layers
ORL: outer retinal layers
PNFL: peripapillary nerve fibre layer
ETDRS: Early Treatment Diabetic Retinopathy Study
VEP: visual evoked potentials flashes
MCV: mean corpuscular volume
MCHC: mean corpuscular haemoglobin concentration
CRP: C reactive protein
LDH: lactate dehydrogenase
SGOT: serum glutamic oxaloacetic transaminase
SGPT: serum glutamic pyruvate transaminase
